# TSH-Mediated TNFα Production in Human Fibrocytes Is Inhibited by Teprotumumab, an IGF-1R Antagonist

**DOI:** 10.1371/journal.pone.0130322

**Published:** 2015-06-18

**Authors:** Hong Chen, Shannon J. C. Shan, Tünde Mester, Yi-Hsuan Wei, Raymond S. Douglas

**Affiliations:** 1 Department of Ophthalmology and Visual Sciences, University of Michigan Medical School, Ann Arbor, MI, United States of America; 2 Department of Ophthalmology of Union Hospital, Tongji Medical College, Huazhong University of Science and Technology, Wuhan, Hubei, People’s Republic of China; 3 Ann Arbor Veterans Administration Medical Center, Ann Arbor, MI, United States of America; Central Michigan University School of Medicine, UNITED STATES

## Abstract

**Purpose:**

Fibrocytes (FC) are bone marrow-derived progenitor cells that are more abundant and infiltrate the thyroid and orbit in Graves orbitopathy (GO). FCs express high levels of thyrotropin receptor (TSHR) and insulin-like growth factor-1 receptor (IGF-1R). These receptors are physically and functionally associated, but their role in GO pathogenesis is not fully delineated. Treatment of FCs with thyroid stimulating hormone (TSH) or M22 (activating antibody to TSHR) induces the production of numerous cytokines, including tumor necrosis factor α (TNFα). Teprotumumab (TMB) is a human monoclonal IGF-1R blocking antibody currently in clinical trial for GO and inhibits TSHR-mediated actions in FCs.

**Aim:**

To characterize the molecular mechanisms underlying TSH-induced TNFα production by FCs, and the role of IGF-1R blockade by TMB.

**Design:**

FCs from healthy and GD patients were treated with combinations of TSH, M22, MG132 and AKTi (inhibitors of NF-κB and Akt, respectively), and TMB. TNFα protein production was measured by Luminex and flow cytometry. Messenger RNA expression was quantified by real time PCR.

**Results:**

Treatment with TSH/M22 induced TNFα protein and mRNA production by FCs, both of which were reduced when FCs were pretreated with MG132 and AKTi (p<0.0001). TMB decreased TSH-induced TNFα protein production in circulating FCs from mean fluorescent index (MFI) value of 2.92 to 1.91, and mRNA expression in cultured FCs from 141- to 52-fold expression (p<0.0001). TMB also decreased M22-induced TNFα protein production from MFI of 1.67 to 1.12, and mRNA expression from 6- to 3-fold expression (p<0.0001).

**Conclusion:**

TSH/M22 stimulates FC production of TNFα mRNA and protein. This process involves the transcription factor NF-κB and its regulator Akt. Blocking IGF-1R attenuates TSH/M22-induced TNFα production. This further delineates the interaction of TSHR and IGF1-R signaling pathways. By modulating the proinflammatory properties of FCs such as TNFα production, TMB may be a promising therapeutic agent for GO.

## Introduction

Fibrocytes are bone marrow-derived progenitor cells of the monocyte lineage [1]. They normally constitute less than 1% of circulating leukocytes [1]. In conditions of inflammation and fibrosis, fibrocytes emerge from the bone marrow and can comprise up to 15% of circulating leukocytes [2–4]. Fibrocytes have a distinct phenotype as they express both leukocyte and fibroblast surface markers [5]. Functionally, fibrocytes have both the proinflammatory properties of leukocytes as well as tissue remodeling capabilities of fibroblasts, making them excellent mediators of inflammation. Fibrocytes migrate to sites of tissue injury in response to chemokines [1, 6, 7] and regulate site-specific inflammation and fibrosis through antigen-specific T cell stimulation [8], cytokine production [9], extracellular matrix remodeling [10], and differentiation into other cell types such as adipocytes and myofibroblasts [11, 12]. Fibrocytes have been implicated in a myriad of inflammatory and fibrotic conditions in the lung [2, 3, 7, 13], liver [14], kidney [15], heart [16], vasculature [17, 18], joints [19], and skin [20, 21]. Accumulating evidence suggests that they also play an important role in the pathogenesis of Graves disease (GD) and Graves orbitopathy (GO).

Graves disease is an autoimmune condition in which autoantibodies bind to the thyrotropin receptor (TSHR) on thyrocytes, leading to increased thyroid hormone production. A subset of patients with GD also develop extrathyroidal manifestations, such as the enlargement of orbital soft tissues as observed in GO. The pathogenesis of GO is incompletely understood [22, 23]. The principal effector cell responsible for the anatomical changes in GO is the orbital fibroblast (OF), which are CD34 positive and analogous to fibrocytes [22, 24, 25]. Two autoantigens seem to be critical for the aberrant activation of OFs in GO: TSHR, and the insulin-like growth factor-1 receptor (IGF-1R) [22, 23]. These two receptors have a close physical and functional relationship. Immunofluorescence and immunoprecipitation studies show that they form a physical complex in thyrocytes and OFs [26]. IGF-1R mediated signaling enhances the cell proliferative effects of TSH or TSHR activating antibodies [27, 28]. On the contrary, interrupting IGF-1R signaling with IGF-1R blocking antibody or a dominant negative receptor mutant can attenuate TSHR downstream signaling in OFs [26, 29]. Interestingly, both of these receptors are overexpressed in fibrocytes [30–32]. Moreover, fibrocytes are more abundant in the peripheral circulation of patients with GD, especially those with severe GO [31]. Together, this suggests that TSHR and IGF-1R signaling in fibrocytes may contribute to the pathogenesis of GO.

Fibrocytes are absent in healthy orbits [31]. However, circulating fibrocytes can infiltrate the thyroid and orbit in GD and GO [31, 32]. Once in the orbit, fibrocytes can differentiate into myofibroblasts and adipocytes, synthesize extracellular matrix proteins, and produce cytokines [12]. A proinflammatory cytokine milieu plays a crucial rule in the activation of OFs [22, 33, 34].

The exuberant production of cytokines by fibrocytes seems to involve TSHR signaling. When treated with TSH or the TSHR activating antibody (M22), which has been shown to be analogous to thyroid stimulating immunoglobulins, fibrocytes produce the cytokines IL-1a, IL-1 receptor antagonist, IL-6, IL-8, IL-12, RANTES, MCP-1, and TNFα [30–32]. Of these cytokines, TNFα is particularly interesting, as its overproduction has been implicated in numerous human diseases [35]. TNFα has a very broad spectrum of biologic effects, such as inducing the production of adhesion molecules and chemokines in fibroblasts and the recruitment of inflammatory cells to local tissues [35]. TNFα is found in the orbital connective tissue of patients with GO, but not in healthy controls, and its levels of expression correlates with the size of extraocular muscles in GO [34, 36]. TNFα antagonists have shown early promise as a treatment option for GO in small cohorts of patients, although no randomized controlled trials have yet been conducted [37–40]. Hence, the production of TNFα in fibrocytes may play a critical role in the pathogenesis of GO.

The mechanisms that stimulate TNFα production in fibrocytes is not known. We had previously shown that TSH/M22-induced IL-6, IL-1RA, and IL-12 production in fibrocytes involves the phosphorylation of protein kinase B (also known as Akt), which activates transcription factors such as NF-κB [30, 41, 42]. Further, we demonstrated that the human monoclonal anti-IGF-1R antibody, teprotumumab (RV001, R1507), attenuates TSH/M22-induced Akt phosphorylation and production of IL-6 and IL-8 in fibrocytes [43]. The present study aims to characterize the molecular mechanisms underlying TSH/M22-induced production of TNFα in fibrocytes. Specifically, we interrogated the NF-κB/Akt pathway and the involvement of the IGF1-R signaling pathway.

## Methods

### Patient samples

Patients with GD (n = 6) were recruited from the Kellogg Eye Center at the University of Michigan. The study was reviewed and approved by the Institutional Review Board of the University of Michigan Health System. Written informed consent was obtained from patients in compliance with policies of the Institutional Review Board of the University of Michigan Health System. Research methods followed the tenets of the Declaration of Helsinki. Healthy controls were obtained from Red Cross filters as described in “Fibrocyte cultures” below.

### Fibrocyte cultures

Fibrocytes were generated from peripheral blood mononuclear cells (PBMC) isolated from the blood of the aforementioned patients with GD or from leukocyte reduction filters provided by the American Red Cross. They were then prepared using conditions as previously described [5, 31, 44]. Briefly, PBMCs were isolated by centrifugation over Ficoll-PaquePlus (catalog #17-1440-03; GE Healthcare Bio-Science). After washing, cells were resuspended in Dulbecco’s Modified Eagle’s Medium (DMEM) supplemented with 10% fetal bovine serum (FBS, Gibco) and 1% penicillin-streptomycin mixture (catalog #15140–122; Life Technologies). Each culture well of a six-well plate was inoculated with 10^7^ cells and incubated at 37C in a 5% CO2 atmosphere. After 7 days, cultures were rinsed, and nonadherent cells were removed by gentle aspiration. Medium was changed every 3 days. After 10 to 14 days of cultivation, culture purity was verified to be >90% fibrocytes by flow cytometry. Twenty-four hours before experimental treatments, medium containing 1% FBS was substituted.

### Flow cytometry

Peripheral blood fibrocytes were assayed within 24 hours of acquisition from the aforementioned patients and healthy controls. Peripheral blood fibrocytes were delineated according to expression of CD45, CD34 and type- I collagen, as previously described [30, 44]. The following anti-human fluorochrome-conjugated mouse antibodies were added: CD45- PERCP (catalog #347464), CD34-APC (catalog #560940), isotype control-FITC (catalog #555748), isotype control-PercP (catalog #340762), and isotype control-APC (catalog # 555751) from BD Biosciences (San Jose, CA), Collagen type-I-FITC (catalog #FCMAB412) from Millipore (Temecula, CA).

### TNFα protein production

Peripheral blood circulating fibrocytes and cultured fibrocytes were treated as indicated in the figure legends with bovine TSH (bTSH, 5mU/ml) from Calbiochem EMD Biosciences (La Jolla, CA), or M22 (1ug/mL, Kronus, Star, ID). In some experiments, cells were pretreated with 500 nM of Akt inhibitor IV (Calbiochem EMD Biosciences, La Jolla, CA), 5 μg/ml of carbobenzoxy-Leu-Leuleucinal (MG132, Cayman, Ann Arbor, MI), or 50 ug/mL of TMB (River Vision Development Corp, NY), for 1 hour before bTSH or M22 stimulation.

Intracellular TNFα in circulating fibrocytes from healthy or GD patients was determined after TSH or M22 stimulation. The PBMCs were treated with Golgi stop (catalog #554724; BD Biosciences) 6 hours after bTSH or M22 stimulation. After 12 hours in culture, the cells were centrifuged (500 x g for 5 minutes), washed and resuspended in PBS (DPBS; Life Technologies, Grand Island, NY) containing 2% FBS with 0.1% sodium azide staining buffer. Surface phenotype selection was performed as above. Fibrocytes were permeabilized and fixed with CytoFix/CytoPerm (catalog # 55472; BD Biosciences) for 20 minutes according to manufacturer instructions and resuspended in 0.1 mL Perm/Wash buffer (catalog #554723; BD Biosciences). Cells were incubated with anti-human TNFα (catalog #340511; BD Biosciences) or isotype control-FITC (cat #555748; BD Biosciences) for 30 minutes. Cells were rinsed and fixed with 1% paraformaldehyde. Analysis was performed using a flow cytometer (LSR II; BD Biosciences). At least 10^6^ events were collected. Mean fluorescent intensity (MFI) was calculated as a ratio of sample geometric mean fluorescence and isotype geometric mean fluorescence. Extracellular TNFα production by cultured fibrocytes was assessed by the Luminex analysis. Fibrocytes were treated as indicated in the figure legends, and the media were subjected to TNFα analysis using human Singleplex Bead kits (LHC3011; Life Technologies).

### TNFα mRNA expression

TNFα mRNA levels were measured by real-time polymerase chain reaction (PCR) following treatment as indicated in the figure legends. Total RNA was isolated from fibrocytes by using Aurum Total RNA Mini Kit (BIO-RAD Laboratories, CA) and reverse transcribed using a reverse transcription kit (Quantitect Reverse Transcription Kit; Qiagen). Quantitative PCR was performed on a thermocycler (CFX96; Bio-Rad Laboratories, Hercules, CA) using a SYBR Green kit from Bio-Rad with the following primers for TNFα: 5’- GTCTCCTACCAGACCAAG-3’ (forward primer); 5’- CAAAGTAGACCTGCCCAGACTC-3’ (reverse primer). Glyceraldehyde-3-phosphate dehydrogenase (GAPDH) was used as the housekeeping gene control, using 5’-TTGCCATCAATGACCCCTT-3’ (forward primer) and 5’-CGCCCCACTTGATTTTGGA-3’ (reverse primer).

### Statistical Analysis

Each experiment was performed in triplicate. Unless otherwise stated, data values are reported as the mean ± standard deviation. Statistical analysis was performed using ANOVA with a confidence level of greater than 95%.

## Results

### TSH and M22 induces TNFα production in healthy and GD fibrocytes

We characterized the effect of TSH or M22 on TNFα production in circulating and cultured fibrocytes. Multiparameter flow cytometry was used to study the circulating fibrocytes, which were defined as the subset of monocytes expressing CD45, type-1 collagen, and CD34. The cultured fibrocytes were derived from PBMCs, which were cultured for 2 weeks into homogeneous and well-differentiated cells.

**Fig 1:**
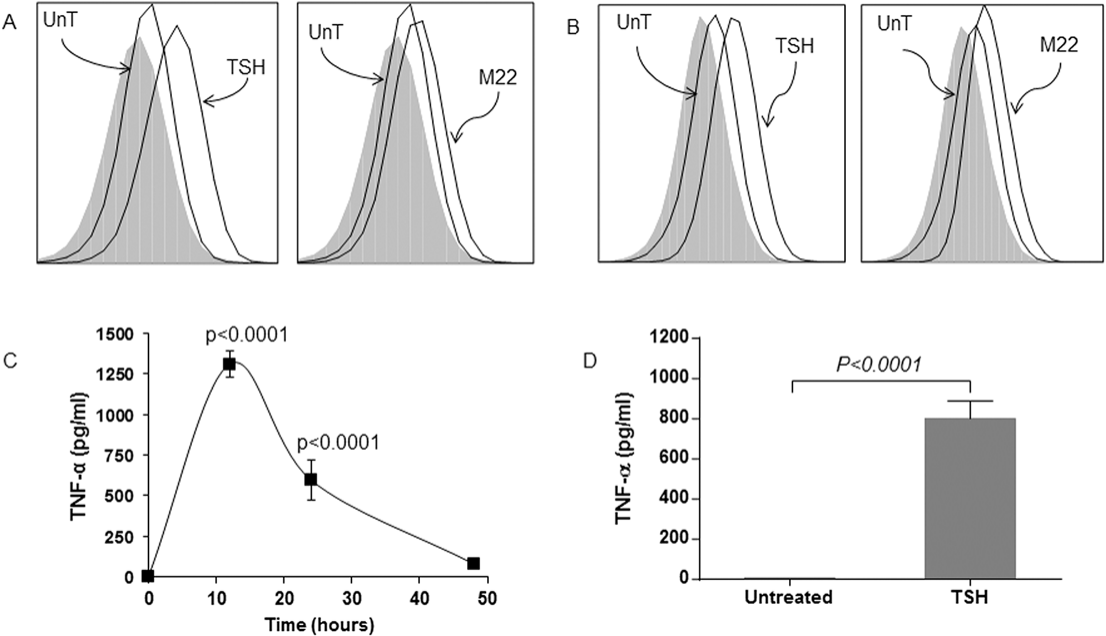
TSH and M22 induce TNFα production in healthy and GD fibrocytes. A representative example of FACS analysis shows that unstimulated circulating fibrocytes from healthy (A) and GD (B) patients produce negligible amount of TNFα (MFI 1.07 and 1.11, respectively). TSH stimulation is associated with significantly higher production of intracellular TNFα (MFI 1.75 and 1.82 for healthy and GD fibrocytes, respectively). M22 treatment of circulating healthy (A) and GD (B) fibrocytes is also associated with increased production of intracellular TNFα (MFI for healthy and GD fibrocytes are 1.54 and 1.52, respectively). Luminex analysis shows that TSH increases extracellular TNFα protein production in cultured healthy (C) and GD (D) fibrocytes. TNFα protein production peaks at 12 hours (1311 pg/ml, p<0.0001) (C). Cultured GD fibrocytes show increased extracellular TNFα production after TSH stimulation (from 8 to 799 pg/ml, p<0.0001) (D).

**Fig 2:**
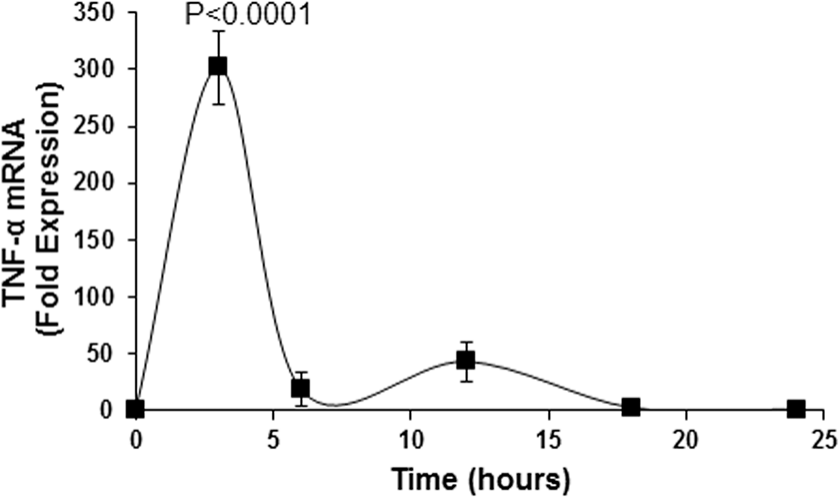
Steady state TNFα mRNA expression after TSH stimulation by real-time PCR in cultured healthy fibrocytes. TNFα mRNA expression is significantly increased in response to TSH stimulation in cultured fibrocytes. The steady state TNFα mRNA expression reaches a peak level at 3 hours (301-fold expression, p<0.0001).

Fluorescence-activated cell sorting analysis shows that unstimulated circulating fibrocytes from healthy (Fig 1A) and GD (Fig 1B) patients produce negligible amount of TNFα (MFI 1.07 and 1.11, respectively). However, TSH stimulation is associated with significantly higher production of intracellular TNFα, as evidenced by the increase in MFI values (MFI 1.75 and 1.82 for healthy and GD fibrocytes, respectively). M22 treatment of circulating healthy (Fig 1A) and GD (Fig 1B) fibrocytes is also associated with increased production of intracellular TNFα (MFI for healthy and GD fibrocytes are 1.54 and 1.52, respectively). Healthy (Fig 1C) and GD (Fig 1D) cultured fibrocytes treated with TSH also show increased extracellular TNFα production. The TNFα protein level in healthy FCs peaks at 12 hours post-treatment (1311 pg/ml, p<0.0001) (Fig 1C). Cultured GD fibrocytes also show increased extracellular TNFα production after TSH stimulation (from 8 to 799 pg/ml, p<0.0001). Moreover, TNFα mRNA expression is significantly increased in response to TSH stimulation in cultured fibrocytes, reaching a peak level at 3 hours (301-fold expression, p<0.0001; Fig 2).

### TSH-induced TNFα production in fibrocytes involves Akt and NF-κB

**Fig 3:**
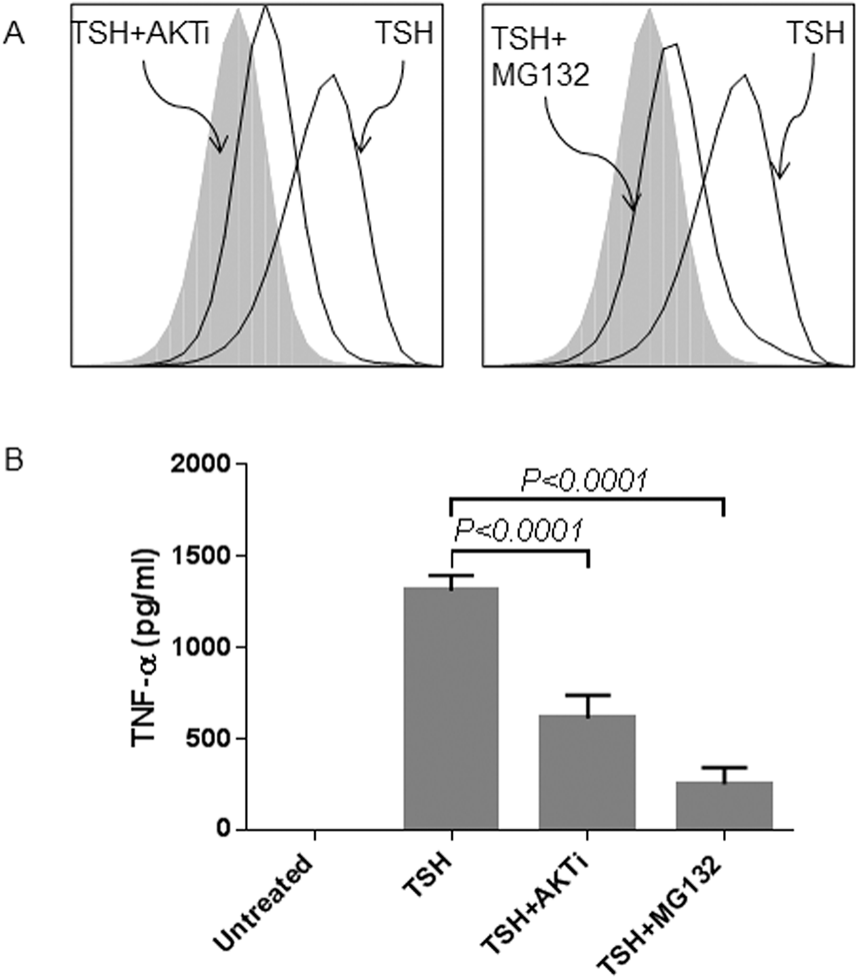
TSH-induced TNFα protein production involves Akt and NF-κB. FACS analysis shows that pretreatment with either AKTi (AKT inhibitor) or MG132 (NF-κB inhibitor) reduces TSH-induced TNFα protein production in healthy circulating fibrocytes (MFI 2.92 reduced to 1.46 with addition of AKTi and 1.33 with MG132) (A). Luminex analysis shows that pretreatment with either AKTi or MG132 reduces TSH-induced TNFα production in healthy cultured fibrocytes. TSH-induced TNFα production of 1312 pg/ml is reduced by 52% to 612 pg/ml with AKTi and by 81% to 251 pg/ml with MG132 (p <0.0001) (B).

**Fig 4:**
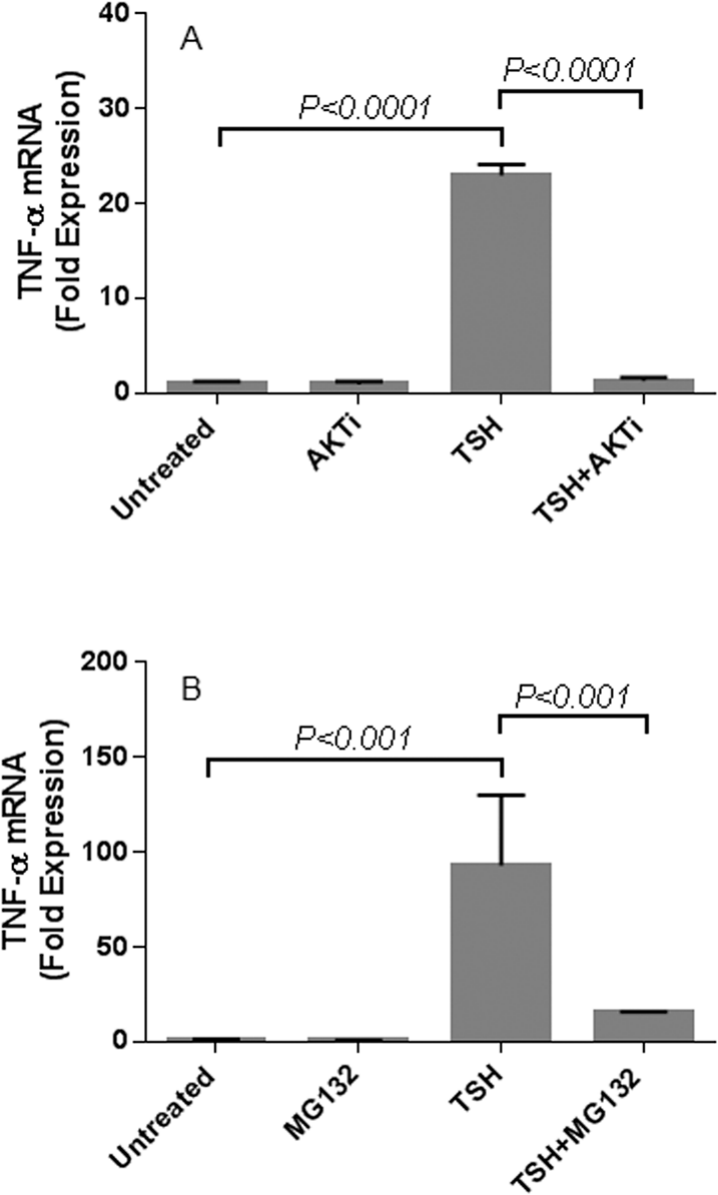
TSH-induced TNFα mRNA expression involves Akt and NF-κB. AKTi (A) or MG132 (B) was added 1 hour before TSH stimulation of healthy cultured fibrocytes. RNA was isolated after 6 hours of induction. AKTi treatment leads to significant reduction in TSH-induced TNFα mRNA expression (from 23-fold to 1.3-fold expression, *p* <0.0001) (A). Similarly, MG132 treatment inhibits TSH-induced TNFα mRNA expression (from 93-fold to 16-fold expression; p< 0.001) (B).

We characterized the role of Akt and NF-κB in TSH-induced TNFα production in fibrocytes by pre-treating the cells with Akt inhibitor IV (AKTi) and NF-κB inhibitor (MG132), respectively (Fig 3). The inhibitors were added 1 hour before TSH stimulation. AKTi diminishes the stimulatory effect of TSH on TNFα production in both circulating (Fig 3A) and cultured fibrocytes (Fig 3B). For circulating fibrocytes, both AKTi and MG132 treatment decrease the amount of TSH-induced TNFα production (MFI 2.92 reduced to 1.46 with addition of AKTi and 1.33 with MG132; Fig 3A). For cultured fibrocytes, TSH-induced TNFα production is reduced by 52% and by 81% after AKTi and MG132 treatment, respectively (TSH-induced TNFα production of 1312 pg/ml is reduced to 612 pg/ml with AKTi and 251 pg/ml with MG132; p <0.0001; Fig 3B). AKTi treatment also leads to significant reduction in TSH-induced TNFα mRNA expression (from 23-fold to 1.3-fold expression, *p* <0.0001; Fig 4A). Similarly, MG132 treatment inhibits TSH-induced TNFα mRNA expression (from 93-fold to 16-fold expression p< 0.001; Fig 4B).

### TSH/M22-induced TNFα production in fibrocytes is attenuated by TMB

**Fig 5:**
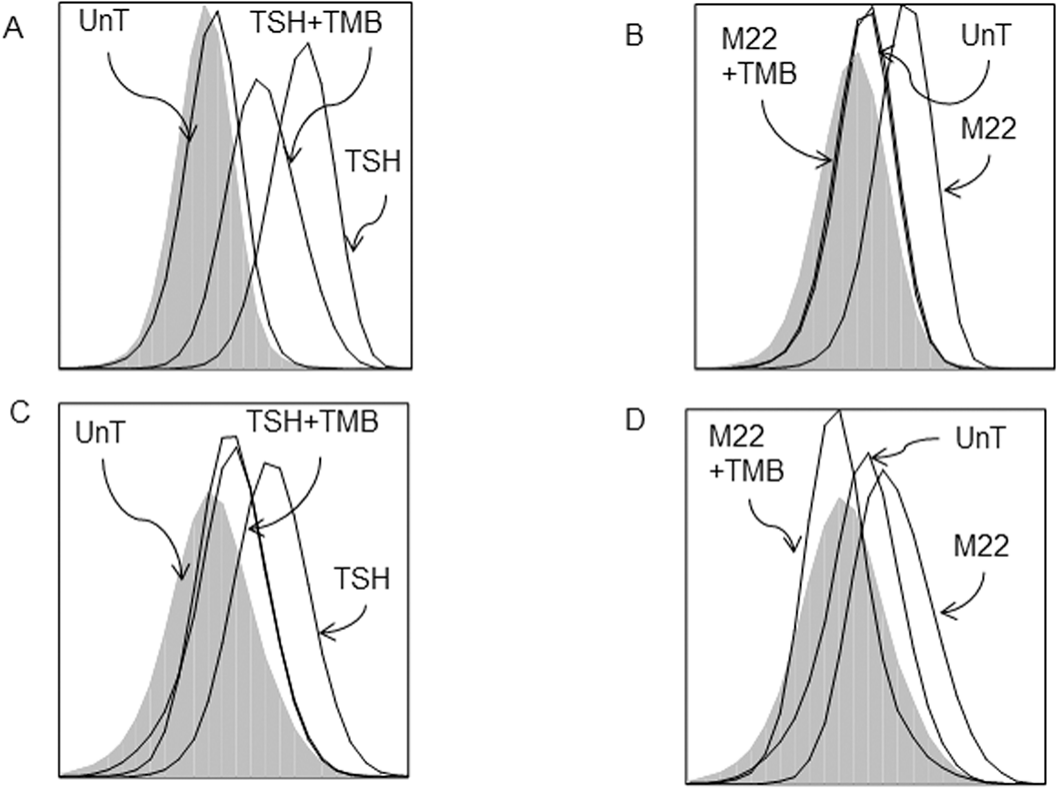
TSH/M22-induced TNFα protein production in fibrocytes is attenuated by TMB, a human anti-IGF-1R monoclonal antibody. Both circulating and cultured fibrocytes were pretreated with TMB, prior to TSH or M22 stimulation. TMB decreases TSH/M22-induced TNFα protein production in healthy (from MFI value of 2.92 to 1.91 for TSH; and from 1.67 to 1.12 for M22) (A, B) and GD circulating fibrocytes (from MFI value of 1.82 to 1.23 for TSH; and from 1.66 to 1.19 for M22) (C, D).

**Fig 6:**
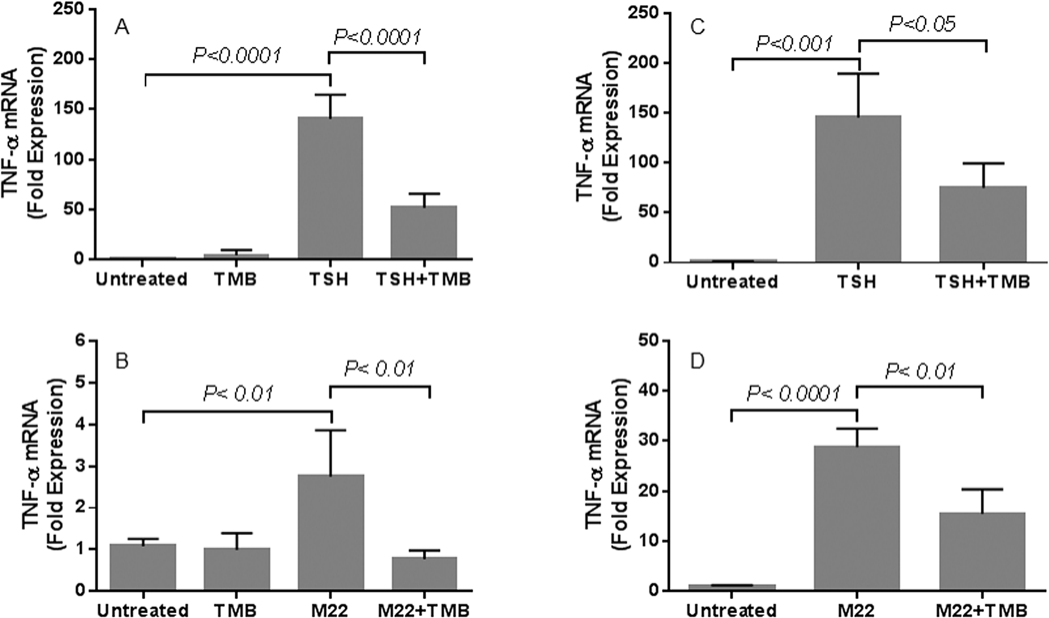
TSH/M22-induced TNFα mRNA expression in fibrocytes is attenuated by TMB. TMB also decreases TSH/M22-induced TNFα mRNA expression in both healthy (from 141- to 52-fold expression for TSH, p<0.0001; and from 6- to 3-fold expression for M22, p< 0.0001) (A, B) and GD cultured fibrocytes (from 145- to 75-fold expression for TSH, p< 0.05; and from 27- to 15-fold expression for M22, p<0.01) (C, D).

We investigated the impact of blocking IGF-1R signaling on TSH/M22-induced TNFα production in fibrocytes. Both circulating and cultured fibrocytes were pretreated with TMB, a human monoclonal anti-IGF-1R antibody, prior to TSH or M22 stimulation. TMB decreases TSH/M22-induced TNFα protein production in healthy (from MFI value of 2.92 to 1.91 for TSH; and from 1.67 to 1.12 for M22) and GD circulating fibrocytes (from MFI value of 1.82 to 1.23 for TSH; and from 1.66 to 1.19 for M22) (Fig 5). TMB also decreases TSH/M22-induced TNFα mRNA expression in cultured healthy (from 141- to 52-fold expression for TSH, p<0.0001; and from 6- to 3-fold expression for M22, p< 0.0001) and GD fibrocytes (from 145- to 75-fold expression for TSH, p< 0.05; and from 27- to 15-fold expression for M22, p<0.01) (Fig 6).

## Discussion

Graves orbitopathy is a disfiguring and potentially vision threatening autoimmune disease. A limited understanding of the pathogenesis of GO has hindered the development of disease-modifying therapeutic agents [22, 23]. Uncovering the cellular and molecular mechanisms underlying the pathogenesis of GO would facilitate the development of more efficacious treatment options. Current evidence points to TSHR and IGF-1R as two autoantigens that play a central role in the pathogenesis of GO. They form a physical complex [26], and are functionally intertwined, as antibodies that block IGF-1R signaling can attenuate TSHR downstream signaling [26, 29]. Both receptors are overexpressed by the fibrocytes, which are bone marrow-derived progenitor cells that infiltrate the orbit in GO. In this study, we demonstrate that TSH/M22 induces the production of TNFα in fibrocytes, and that this stimulatory effect is partially dependent on the IGF-1R signaling pathway and can be attenuated by the anti-IGF-1R monoclonal antibody, TMB.

Unstimulated fibrocytes express negligible TNFα. TSH or M22 induces significant levels of TNFα in both healthy and GD fibrocytes. Fibrocytes are absent in healthy orbits but infiltrate the orbit in GO [31]. Interestingly, immunohistochemical evidence shows that TNFα is also present only in orbits with GO, and that it is most likely derived from infiltrating mononuclear cells [36]. While many cell types can produce TNFα, its principal source is monocytes and macrophages [35, 45]. Therefore, fibrocytes, which are of the monocyte lineage, may be a key source of TNFα production in GO once they infiltrate the orbit.

TNFα is a potent proinflammatory cytokine. Its overproduction has been postulated to play an initiatory role in inflammation by triggering a cytokine cascade in several human diseases [46, 47]. Our findings show that TSH/M22-induced TNFα production peaks at 3 hours for mRNA expression, and at 12 hours for protein production. This is much earlier than the effects observed for other cytokines: TSH/M22-induced IL-6 and IL-8 mRNA expression peaks at 12 to 24 hours [30]. The significance of these findings needs to be further investigated. Nevertheless, our observations may be in line with other evidence suggesting that TNFα is at the top of a cytokine cascade. Therapeutic agents that target either TNFα itself (TNFα antagonists) or the mechanisms triggering TNFα production in fibrocytes may interrupt the propagation of the inflammatory response in GO. Anecdotal evidence suggests that the former may be associated with clinical improvement in GO, although no randomized controlled trials have been conducted [37–40]. To facilitate the development of the latter, this study aimed to understand the mechanisms underlying TNFα production in fibrocytes.

Our findings suggest that TSH-induced TNFα production may be mediated at the transcriptional level in fibrocytes. Both TNFα steady state mRNA and protein levels are increased in response to TSH, and both are diminished when the fibrocytes are pretreated with inhibitors of NF-κB and Akt. Therefore, these transcription regulators appear to play a role in TSH-induced transcriptional upregulation of TNFα in fibrocytes. This is consistent with our previous results showing that both NF-κB and Akt are involved in TSH-induced IL-1RA, IL-6, and IL-12 production in fibrocytes [30, 41, 42]. Also in line with our current findings is the observation that the proximal promoter of the TNFα gene contains a binding site for NF-κB [48]. Therefore, we confirm that TSH-induced cytokine production in fibrocytes involves the NF-κB and Akt pathway.

The IGF-1R signaling pathway also plays a crucial role in TSH/M22-induced TNFα production in fibrocytes. Pretreatment of the cells with the anti-IGF-1R antibody, TMB, attenuates the stimulatory effects of TSH/M22 on TNFα production. This is in agreement with our previous studies demonstrating the inhibitory effect of TMB on TSH-induced IL-6 and IL-8 production and TSH-induced Akt phosphorylation in fibrocytes [43]. Indeed, TMB can also exerts its influence by reducing TSHR and IGF-1R display on fibrocytes [43]. Collectively, the aforementioned evidence serve as the rationale for investigating the role of blocking IGF-1R signaling pathway in the treatment of GO. A multicenter, placebo-controlled, phase II clinical trial (NCT01868997) is currently underway to assess the efficacy of TMB in treating moderate-to-severe active GO.

Our current findings suggest that TMB may be a promising disease-modifying therapy for GO. They further support the model that TSHR and IGF-1R have a functional relationship in the pathogenesis in GO. However, it should be noted that TMB does not completely abolish the stimulatory effect of TSH/M22 on TNFα production. Hence, part of TSHR signaling is likely IGF-1R independent. Future experiments should systemically delineate the complex interplay between the signaling pathways of these two receptors in fibrocytes. This would advance our understanding of the pathogenesis of GO and facilitate the discovery of novel therapeutic agents.

## Conclusion

Accumulating evidence supports an important role of the fibrocytes in the pathogenesis of GO. They infiltrate the orbit in GO and can be activated to produce numerous proinflammatory cytokines, thereby influencing site-specific tissue reactivity and propagating the inflammatory response. In this study, we show that the fibrocytes may be a principal source of TNFα, a key proinflammatory cytokine, in GO. TSH/M22 treatment in the fibrocytes may lead to transcriptional upregulation of TNFα, which involves the transcriptional regulators NFkB and Akt. TMB can attenuate TSH/M22-induced TNFα production in fibrocytes. Therefore, we provide *in vitro* evidence that TMB can modulate the proinflammatory properties of fibrocytes, and is a promising therapeutic agent for GO.

## References

[1] Chesney J, Bucala R. Peripheral blood fibrocytes: mesenchymal precursor cells and the pathogenesis of fibrosis. Current rheumatology reports. 2000;2(6):501–5. . 11123104 10.1007/s11926-000-0027-5

[2] Mehrad B, Burdick MD, Zisman DA, Keane MP, Belperio JA, Strieter RM. Circulating peripheral blood fibrocytes in human fibrotic interstitial lung disease. Biochemical and biophysical research communications. 2007;353(1):104–8. 10.1016/j.bbrc.2006.11.149 . 17174272

[3] Moeller A, Gilpin SE, Ask K, Cox G, Cook D, Gauldie J, et al Circulating fibrocytes are an indicator of poor prognosis in idiopathic pulmonary fibrosis. American journal of respiratory and critical care medicine. 2009;179(7):588–94. 10.1164/rccm.200810-1534OC . 19151190

[4] Herzog EL, Bucala R. Fibrocytes in health and disease. Experimental hematology. 2010;38(7):548–56. 10.1016/j.exphem.2010.03.004 20303382 PMC3136351

[5] Bucala R, Spiegel LA, Chesney J, Hogan M, Cerami A. Circulating fibrocytes define a new leukocyte subpopulation that mediates tissue repair. Molecular medicine. 1994;1(1):71–81. 8790603 PMC2229929

[6] Strieter RM, Gomperts BN, Keane MP. The role of CXC chemokines in pulmonary fibrosis. The Journal of clinical investigation. 2007;117(3):549–56. 10.1172/JCI30562 17332882 PMC1804376

[7] Phillips RJ, Burdick MD, Hong K, Lutz MA, Murray LA, Xue YY, et al Circulating fibrocytes traffic to the lungs in response to CXCL12 and mediate fibrosis. The Journal of clinical investigation. 2004;114(3):438–46. 10.1172/JCI20997 15286810 PMC484979

[8] Chesney J, Bacher M, Bender A, Bucala R. The peripheral blood fibrocyte is a potent antigen-presenting cell capable of priming naive T cells in situ. Proceedings of the National Academy of Sciences of the United States of America. 1997;94(12):6307–12. 9177213 10.1073/pnas.94.12.6307 PMC21045

[9] Hartlapp I, Abe R, Saeed RW, Peng T, Voelter W, Bucala R, et al Fibrocytes induce an angiogenic phenotype in cultured endothelial cells and promote angiogenesis in vivo. FASEB journal: official publication of the Federation of American Societies for Experimental Biology. 2001;15(12):2215–24. 10.1096/fj.01-0049com . 11641248

[10] Bianchetti L, Barczyk M, Cardoso J, Schmidt M, Bellini A, Mattoli S. Extracellular matrix remodelling properties of human fibrocytes. Journal of cellular and molecular medicine. 2012;16(3):483–95. 10.1111/j.1582-4934.2011.01344.x . 21595824 PMC3822925

[11] Abe R, Donnelly SC, Peng T, Bucala R, Metz CN. Peripheral blood fibrocytes: differentiation pathway and migration to wound sites. Journal of immunology. 2001;166(12):7556–62. . 11390511 10.4049/jimmunol.166.12.7556

[12] Hong KM, Belperio JA, Keane MP, Burdick MD, Strieter RM. Differentiation of human circulating fibrocytes as mediated by transforming growth factor-beta and peroxisome proliferator-activated receptor gamma. The Journal of biological chemistry. 2007;282(31):22910–20. 10.1074/jbc.M703597200 . 17556364

[13] Schmidt M, Sun G, Stacey MA, Mori L, Mattoli S. Identification of circulating fibrocytes as precursors of bronchial myofibroblasts in asthma. Journal of immunology. 2003;171(1):380–9. . 12817021 10.4049/jimmunol.171.1.380

[14] Kisseleva T, Uchinami H, Feirt N, Quintana-Bustamante O, Segovia JC, Schwabe RF, et al Bone marrow-derived fibrocytes participate in pathogenesis of liver fibrosis. Journal of hepatology. 2006;45(3):429–38. 10.1016/j.jhep.2006.04.014 . 16846660

[15] Sakai N, Wada T, Yokoyama H, Lipp M, Ueha S, Matsushima K, et al Secondary lymphoid tissue chemokine (SLC/CCL21)/CCR7 signaling regulates fibrocytes in renal fibrosis. Proceedings of the National Academy of Sciences of the United States of America. 2006;103(38):14098–103. 10.1073/pnas.0511200103 16966615 PMC1599918

[16] Haudek SB, Xia Y, Huebener P, Lee JM, Carlson S, Crawford JR, et al Bone marrow-derived fibroblast precursors mediate ischemic cardiomyopathy in mice. Proceedings of the National Academy of Sciences of the United States of America. 2006;103(48):18284–9. 10.1073/pnas.0608799103 17114286 PMC1643845

[17] Zulli A, Buxton BF, Black MJ, Hare DL. CD34 Class III positive cells are present in atherosclerotic plaques of the rabbit model of atherosclerosis. Histochemistry and cell biology. 2005;124(6):517–22. 10.1007/s00418-005-0072-2 . 16177890

[18] Varcoe RL, Mikhail M, Guiffre AK, Pennings G, Vicaretti M, Hawthorne WJ, et al The role of the fibrocyte in intimal hyperplasia. Journal of thrombosis and haemostasis: JTH. 2006;4(5):1125–33. 10.1111/j.1538-7836.2006.01924.x . 16689767

[19] Galligan CL, Fish EN. Circulating fibrocytes contribute to the pathogenesis of collagen antibody-induced arthritis. Arthritis and rheumatism. 2012;64(11):3583–93. 10.1002/art.34589 . 22729466

[20] Wang J, Jiao H, Stewart TL, Shankowsky HA, Scott PG, Tredget EE. Improvement in postburn hypertrophic scar after treatment with IFN-alpha2b is associated with decreased fibrocytes. Journal of interferon & cytokine research: the official journal of the International Society for Interferon and Cytokine Research. 2007;27(11):921–30. 10.1089/jir.2007.0008 . 18052725

[21] Yang L, Scott PG, Dodd C, Medina A, Jiao H, Shankowsky HA, et al Identification of fibrocytes in postburn hypertrophic scar. Wound repair and regeneration: official publication of the Wound Healing Society [and] the European Tissue Repair Society. 2005;13(4):398–404. 10.1111/j.1067-1927.2005.130407.x . 16008729

[22] Shan SJ, Douglas RS. The pathophysiology of thyroid eye disease. Journal of neuro-ophthalmology: the official journal of the North American Neuro-Ophthalmology Society. 2014;34(2):177–85. 10.1097/WNO.0000000000000132 . 24821101

[23] Wang Y, Smith TJ. Current concepts in the molecular pathogenesis of thyroid-associated ophthalmopathy. Investigative ophthalmology & visual science. 2014;55(3):1735–48. 10.1167/iovs.14-14002 24651704 PMC3968932

[24] Smith RS, Smith TJ, Blieden TM, Phipps RP. Fibroblasts as sentinel cells. Synthesis of chemokines and regulation of inflammation. The American journal of pathology. 1997;151(2):317–22. 9250144 PMC1858004

[25] Smith TJ, Tsai CC, Shih MJ, Tsui S, Chen B, Han R, et al Unique attributes of orbital fibroblasts and global alterations in IGF-1 receptor signaling could explain thyroid-associated ophthalmopathy. Thyroid. 2008;18(9):983–8. 10.1089/thy.2007.0404 . 18788919 PMC2574420

[26] Tsui S, Naik V, Hoa N, Hwang CJ, Afifiyan NF, Sinha Hikim A, et al Evidence for an association between thyroid-stimulating hormone and insulin-like growth factor 1 receptors: a tale of two antigens implicated in Graves' disease. Journal of immunology. 2008;181(6):4397–405. 18768899 10.4049/jimmunol.181.6.4397 PMC2775538

[27] Tramontano D, Cushing GW, Moses AC, Ingbar SH. Insulin-like growth factor-I stimulates the growth of rat thyroid cells in culture and synergizes the stimulation of DNA synthesis induced by TSH and Graves'-IgG. Endocrinology. 1986;119(2):940–2. 10.1210/endo-119-2-940 . 2874015

[28] Clement S, Refetoff S, Robaye B, Dumont JE, Schurmans S. Low TSH requirement and goiter in transgenic mice overexpressing IGF-I and IGF-Ir receptor in the thyroid gland. Endocrinology. 2001;142(12):5131–9. 10.1210/endo.142.12.8534 . 11713206

[29] Kumar S, Iyer S, Bauer H, Coenen M, Bahn RS. A stimulatory thyrotropin receptor antibody enhances hyaluronic acid synthesis in graves' orbital fibroblasts: inhibition by an IGF-I receptor blocking antibody. The Journal of clinical endocrinology and metabolism. 2012;97(5):1681–7. 10.1210/jc.2011-2890 22399503 PMC3339886

[30] Gillespie EF, Papageorgiou KI, Fernando R, Raychaudhuri N, Cockerham KP, Charara LK, et al Increased expression of TSH receptor by fibrocytes in thyroid-associated ophthalmopathy leads to chemokine production. The Journal of clinical endocrinology and metabolism. 2012;97(5):E740–6. 10.1210/jc.2011-2514 22399514 PMC3339887

[31] Douglas RS, Afifiyan NF, Hwang CJ, Chong K, Haider U, Richards P, et al Increased generation of fibrocytes in thyroid-associated ophthalmopathy. The Journal of clinical endocrinology and metabolism. 2010;95(1):430–8. 10.1210/jc.2009-1614 19897675 PMC2805489

[32] Smith TJ, Padovani-Claudio DA, Lu Y, Raychaudhuri N, Fernando R, Atkins S, et al Fibroblasts expressing the thyrotropin receptor overarch thyroid and orbit in Graves' disease. The Journal of clinical endocrinology and metabolism. 2011;96(12):3827–37. 10.1210/jc.2011-1249 21956421 PMC3232631

[33] Gianoukakis AG, Khadavi N, Smith TJ. Cytokines, Graves' disease, and thyroid-associated ophthalmopathy. Thyroid. 2008;18(9):953–8. Epub 2008/08/21. 10.1089/thy.2007.0405 18713026 PMC2879490

[34] Hiromatsu Y, Yang D, Bednarczuk T, Miyake I, Nonaka K, Inoue Y. Cytokine profiles in eye muscle tissue and orbital fat tissue from patients with thyroid-associated ophthalmopathy. The Journal of clinical endocrinology and metabolism. 2000;85(3):1194–9. 10.1210/jcem.85.3.6433 . 10720061

[35] Vassalli P. The pathophysiology of tumor necrosis factors. Annual review of immunology. 1992;10:411–52. 10.1146/annurev.iy.10.040192.002211 . 1590993

[36] Heufelder AE, Bahn RS. Detection and localization of cytokine immunoreactivity in retro-ocular connective tissue in Graves' ophthalmopathy. European journal of clinical investigation. 1993;23(1):10–7. . 8444271 10.1111/j.1365-2362.1993.tb00712.x

[37] Durrani OM, Reuser TQ, Murray PI. Infliximab: a novel treatment for sight-threatening thyroid associated ophthalmopathy. Orbit. 2005;24(2):117–9. 10.1080/01676830590912562 . 16191800

[38] Paridaens D, van den Bosch WA, van der Loos TL, Krenning EP, van Hagen PM. The effect of etanercept on Graves' ophthalmopathy: a pilot study. Eye. 2005;19(12):1286–9. 10.1038/sj.eye.6701768 . 15550932

[39] Prummel MF, Mourits MP, Berghout A, Krenning EP, van der Gaag R, Koornneef L, et al Prednisone and cyclosporine in the treatment of severe Graves' ophthalmopathy. The New England journal of medicine. 1989;321(20):1353–9. 10.1056/NEJM198911163212002 . 2519530

[40] Ayabe R, Rootman DB, Hwang CJ, Ben-Artzi A, Goldberg R. Adalimumab as steroid-sparing treatment of inflammatory-stage thyroid eye disease. Ophthalmic plastic and reconstructive surgery. 2014;30(5):415–9. 10.1097/IOP.0000000000000211 . 24978425

[41] Raychaudhuri N, Fernando R, Smith TJ. Thyrotropin regulates IL-6 expression in CD34+ fibrocytes: clear delineation of its cAMP-independent actions. PloS one. 2013;8(9):e75100 10.1371/journal.pone.0075100 24086448 PMC3783445

[42] Li B, Smith TJ. PI3K/AKT pathway mediates induction of IL-1RA by TSH in fibrocytes: modulation by PTEN. The Journal of clinical endocrinology and metabolism. 2014;99(9):3363–72. 10.1210/jc.2014-1257 24840811 PMC4154109

[43] Chen H, Mester T, Raychaudhuri N, Kauh CY, Gupta S, Smith TJ, et al Teprotumumab, an IGF-1R blocking monoclonal antibody inhibits TSH and IGF-1 action in fibrocytes. The Journal of clinical endocrinology and metabolism. 2014;99(9):E1635–40. 10.1210/jc.2014-1580 24878056 PMC4154099

[44] Gillespie EF, Raychaudhuri N, Papageorgiou KI, Atkins SJ, Lu Y, Charara LK, et al Interleukin-6 production in CD40-engaged fibrocytes in thyroid-associated ophthalmopathy: involvement of Akt and NF-kappaB. Investigative ophthalmology & visual science. 2012;53(12):7746–53. 10.1167/iovs.12-9861 23092922 PMC3506052

[45] MacNaul KL, Hutchinson NI, Parsons JN, Bayne EK, Tocci MJ. Analysis of IL-1 and TNF-alpha gene expression in human rheumatoid synoviocytes and normal monocytes by in situ hybridization. Journal of immunology. 1990;145(12):4154–66. . 2258613

[46] Brennan FM, Chantry D, Jackson A, Maini R, Feldmann M. Inhibitory effect of TNF alpha antibodies on synovial cell interleukin-1 production in rheumatoid arthritis. Lancet. 1989;2(8657):244–7. . 2569055 10.1016/s0140-6736(89)90430-3

[47] Terrando N, Monaco C, Ma D, Foxwell BM, Feldmann M, Maze M. Tumor necrosis factor-alpha triggers a cytokine cascade yielding postoperative cognitive decline. Proceedings of the National Academy of Sciences of the United States of America. 2010;107(47):20518–22. 10.1073/pnas.1014557107 21041647 PMC2996666

[48] Goldfeld AE, Doyle C, Maniatis T. Human tumor necrosis factor alpha gene regulation by virus and lipopolysaccharide. Proceedings of the National Academy of Sciences of the United States of America. 1990;87(24):9769–73. 2263628 10.1073/pnas.87.24.9769 PMC55255

